# Research Progress of Natural Rubber Wet Mixing Technology

**DOI:** 10.3390/polym16131899

**Published:** 2024-07-02

**Authors:** Qinghan Zhao, Fangyan Niu, Junyu Liu, Haishan Yin

**Affiliations:** College of Electromechanical and Engineering, Qingdao University of Science and Technology, Qingdao 266100, China; zqhqust@163.com (Q.Z.); nfy5068@163.com (F.N.); ljy38324@126.com (J.L.)

**Keywords:** wet compounding, fillers, natural rubber, research progress, rubber reinforcement, composites

## Abstract

The performance of natural rubber (NR), a naturally occurring and sustainable material, can be greatly enhanced by adding different fillers to the NR matrix. The homogeneous dispersion of fillers in the NR matrix is a key factor in their ability to reinforce. As a novel method, wet mixing technology may effectively provide good filler dispersion in the NR matrix while overcoming the drawbacks of conventional dry mixing. This study examines the literature on wet mixing fillers, such as graphene, carbon nanotubes, silica, carbon black, and others, to prepare natural rubber composites. It also focuses on the wet preparation techniques and key characteristics of these fillers. Furthermore, the mechanism of filler reinforcement is also examined. To give guidance for the future development of wet mixing technology, this study also highlights the shortcomings of the current system and the urgent need to address them.

## 1. Introduction

Extracted from the Brazilian rubber tree, natural rubber (NR) is a sustainable and renewable resource. Because of its exceptional physical qualities, including its exceptional elasticity, resilience, high tensile strength, and raw strength, it is one of the elastomers that is utilized in tire production the most. Because of its low heat buildup, resistance to fracture formation, and low hysteresis, NR is favored for tire production [1]. In most cases, the qualities of pure rubber are insufficient for practical uses; therefore, reinforcing fillers must be added to improve the properties of rubber [2]. The failure behavior of rubber composites is greatly influenced by the reinforcing fillers’ particle size, form factor, surface activity, number of filler components, and dispersion condition in the rubber matrix [3].

Traditional mixing, sometimes referred to as dry mixing, is mostly accomplished by alternating or continuously mixing fillers with granular or solid rubber ingredients. Reinforcing fillers, such as carbon black, tend to clump when traditional dry mixing techniques are applied. This leads to inadequate filler dispersion in the rubber matrix, and the resulting rubber composite does not show the desired qualities.

The wet mixing technique is a novel approach to chemical solidification that involves combining filler solution and polymer latex (solution) following conventional dry mixing. Wet mixing can address issues with dust pollution, energy consumption, and other issues associated with traditional dry mixing since it uses a liquid phase to complete the mixing and dispersion of the filler and rubber. Wet mixing offers notable benefits, particularly for heavily packed rubber composites.

**Figure 1:**
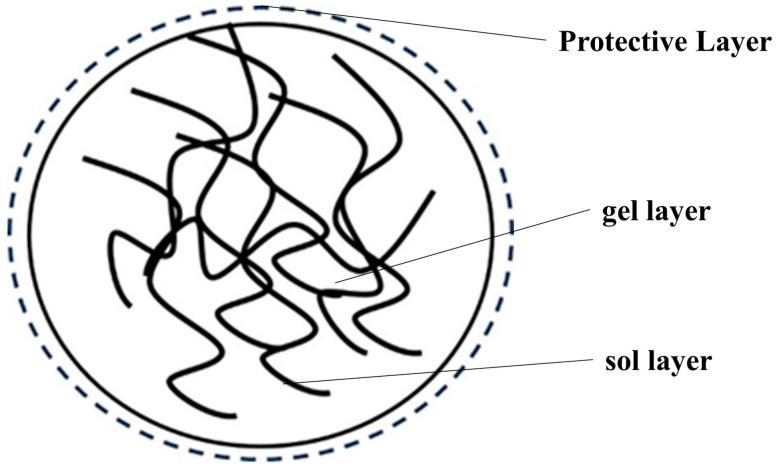
Schematic structure of natural latex rubber particles.

According to contemporary basic research on rubber composites, in order to manufacture rubber composites with outstanding overall performance, a thorough investigation of the issue of particle binding at the microscopic level is required. As illustrated in Figure 1, the three layers that make up natural latex’s rubber particles are a viscous sol-gel layer made of rubber hydrocarbons with a small degree of polymerization in the innermost layer, a gel layer made of rubber hydrocarbons with a large degree of polymerization in the middle layer, and a protective layer made of proteins and lipids in the outermost layer. Of these, the protective layer can help the rubber particles stay evenly distributed in the natural latex; however, because of its presence, it prevents the filler particles from coming into direct contact with the rubber particles in the natural latex, which in turn impacts the filler reinforcement’s effectiveness.

The filler particles must first have a high dispersibility to effectuate the reinforcement of natural latex by reinforcing filler in an emulsion condition and to obtain an outstanding reinforcing effect. To produce a uniform distribution of fillers in the natural latex film and improve the reinforcing effect, co-precipitation of the natural latex particles and reinforcing filler particles is required when coalescence occurs. Rubber hydrocarbon and reinforcing filler need to come into direct contact with one another; the greater their mutual contact area, the better and more excellent the reinforcing effect.

This research examines the wet fabrication and application of graphene (GO)/NR, carbon nanotubes (CNTs)/NR, silica (SiO_2_)/NR, and carbon black (CB)/NR composites. While there are a lot of reviews on natural rubber composites, there are not many on the wet preparation of NR composites. This review aims to close that gap by offering a thorough analysis of the body of research as well as a forecast for the promising future possibilities in this area.

## 2. Wet Preparation of NR Composites

The two primary challenges of agglomeration and filler–matrix interaction must be overcome to produce NR composites with homogenous filler dispersion. Stronger filler–matrix contact and high filler dispersion are necessary for the composites to have a reinforcing effect. The three main wet preparation techniques for NR composites are sol-gel, latex, and solution mixing.

### 2.1. Latex Mixing Method

This approach uses natural latex as the matrix and stabilizes the reinforcing particles through a series of techniques that culminate in a homogenous mixture and agglomeration. Zhao et al. [4] prepared GO/NR nanocomposites by using the latex blending method. It was discovered that the latex mixing approach may be used to accomplish the homogenous dispersion of GO in the NR matrix. Without compromising the final strength, the uniformly distributed GO greatly raised the tensile strength and energy storage modulus of NR at a reduced filling percentage. This process is less harmful to the environment than the solution mixing method since it does not require costly and highly toxic organic solvents [5].

### 2.2. Solution Mixing Method

By combining the rubber solution and nanofiller dispersion and then draining the solvent, rubber composites were produced using this technique [6]. Yang et al. [7] prepared SiO_2_/NR composites by accomplishing the mixing between silica and rubber molecular chains in an organic phase solution. The silica/natural rubber composites made via the solution approach presented superior filler dispersion when compared to those made by the dry mixing method. The main disadvantages of this technique, however, are its high cost, the need for a lot of solvents during the solvent removal process, and the environmental issues related to disposing of solvents [8]. The final properties of the rubber composites are greatly influenced by the solvent used during the preparation process [5].

### 2.3. Sol-Gel Method

Precursors like siloxanes or metal salts are added to the rubber matrix in the sol-gel process to facilitate hydrolysis and condensation processes, which produce uniformly distributed nanoparticles in situ. Tetraethoxysilane (TEOS) is a widely utilized precursor for the preparation of SiO_2_/NR composites. Poompradub et al. [9] used ethyl orthosilicate (TEOS) as a silica precursor to create SiO_2_/NR composites utilizing the sol-gel process. When compared to commercial (ectopic formation) SiO_2_/NR vulcanizates, the mechanical and thermal characteristics of SiO_2_/NR composites, including silica produced in situ using the sol-gel method, were greatly enhanced. While the non-in situ generated commercial silica agglomerated in the NR matrix, the in situ-created silica particles were evenly dispersed throughout the matrix.

## 3. Progress in the Application of Wet Mixing Process in Natural Rubber Composites

### 3.1. Carbon Black (CB)/Natural Rubber Composites

#### 3.1.1. Mechanism of Carbon Black Reinforced Rubber

Carbon black is thought to be the most efficient filler material additive since it makes rubber-based polymers stronger and harder [10]. The binding effect between the carbon black particles and the rubber matrix is the primary mechanism by which carbon black is reinforced with rubber. Due to the small particle size of carbon black, its large contact area with the rubber matrix, and its ease of infiltration by rubber molecules, physical bonding mostly manifests as van der Waals force. Simultaneously, the presence of active sites on the surface of carbon black particles, which have a higher surface energy and are easier to firmly connect with the rubber matrix, is the primary cause of chemical adsorption. Numerous academics and researchers have investigated the workings of carbon black reinforcing rubber in great detail, and they have put forth the following models of reinforcement.

According to the principle of volumetric effect, scattered carbon black in the rubber matrix creates a solvent effect that raises the rubber’s viscosity and has a strong reinforcing impact. The majority of the mechanical characteristics of carbon black rubber composites are associated with rubber’s tendency to increase viscosity. Rubber’s mechanical strength is increased because the molecular chains adsorbed on the surface of carbon black particles exhibit an orientated state. When there is a tensile tension applied to the rubber, if the carbon black adsorption on the rubber molecular chain is weak, the “gap phenomenon” will occur [11]. Rubber can more easily penetrate areas with higher carbon black particle surface adsorption energies. The degree of surface structure of carbon black can be improved by actively treating the particles’ surfaces, which increases the material’s ability to permeate the rubber matrix and distribute itself evenly.

However, the surface structure theory [12] contends that the active filler’s surface is not smooth and that the filler’s size range and surface roughness would affect the rubber’s characteristics. Donnet et al. [13] first saw the surface of carbon black particles using a scanning tunneling electron microscope. They discovered that the surface of the carbon black is uneven and rough, with sharp edges separating the particles. Based on these observations, they proposed a model theory for carbon black. Later, Donnet et al. discovered numerous localized crystal structures on carbon black’s surface and put forth the notion of the polymer adsorption carbon black surface model. 

A molecular chain sliding model was proposed by Dannenberg et al. [14] in which diverse adsorption activities are exhibited by carbon black particles and rubber macromolecules gliding over the surface. Under tension, the rubber chains adsorbed on the carbon black surface lengthen and slide. Hysteresis energy loss into thermal energy occurs during the molecular chain sliding process, preventing the rubber substance from being destroyed. Most molecular chains do not require sliding when stretched back to their initial length, demonstrating the phenomena of stress relaxation.

The interaction between the filler and the rubber is crucial for the reinforcing of filled rubbers, as several investigations have demonstrated. Generally speaking, the quantity of binding gel that forms between the filler and the rubber is how much of an interaction there is. The inclusive rubber model, the rubber-shell model, and the glassy rubber-shell model are three of the most developed theoretical models of binding gel that have been put forth.

**Figure 2:**
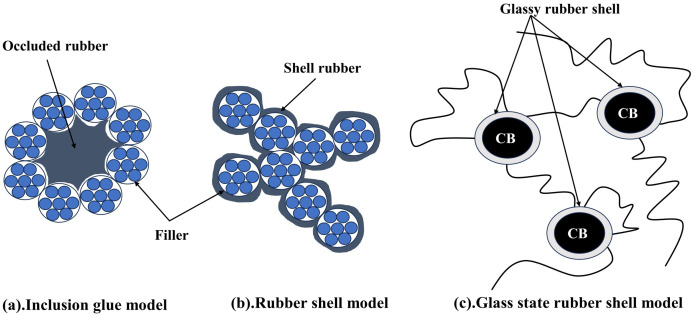
Binding gel theory model diagram.

The inclusive rubber model, as described by Medalia [15] and Kraus [16], states that a certain amount of rubber is contained in the spaces of the filler network, whereas aggregates or agglomerates of active fillers can create a network of fillers due to their adsorption (Figure 2a). Due to its decreased mobility, the encapsulated rubber no longer contributes to the rubber’s elastic behavior and joins the packing, increasing the packing’s effective volume.

**Figure 3:**
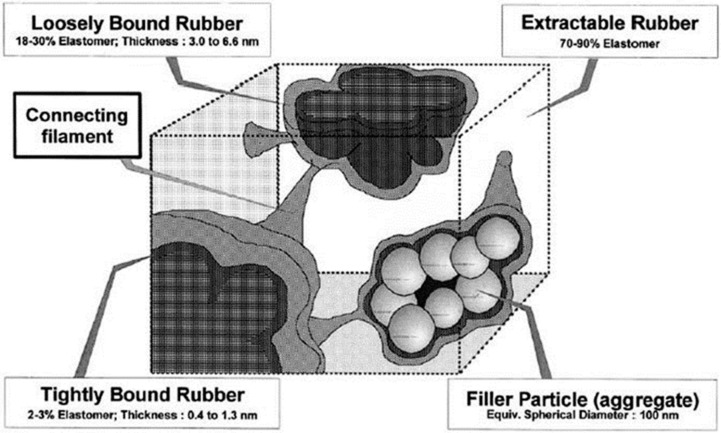
Theoretical diagram of rubber layer distribution around carbon black particles [20].

Rubber macromolecular chains may be chemisorbed by the filler’s surface active sites as a result of chemisorption around the filler particles, as demonstrated in Figure 2b, according to the rubber shell model put out by Smit [17] and Pliskin [18]. Using Figure 2c as support, O’Brien [19] further suggested that the rubber molecular chains adsorbed around the filler particles are practically glassy and have no motility. In this regard, LeBlanc [20] conducted numerous experimental investigations utilizing H1-NMR, and he suggested that rubber adsorbed in the immediate area of the filler particles as a layer that was strongly linked. The external force field is in a glassy state and does not affect the macromolecular chains, which interact significantly with the filler particles. When the adsorption force weakens, rubber molecule chains become more mobile, and the binding layer becomes comparatively free. As seen in Figure 3, the rubber’s solvent can dissolve the opposing outer layer, or free rubber, which is not constrained by the filler particles.

**Figure 4:**
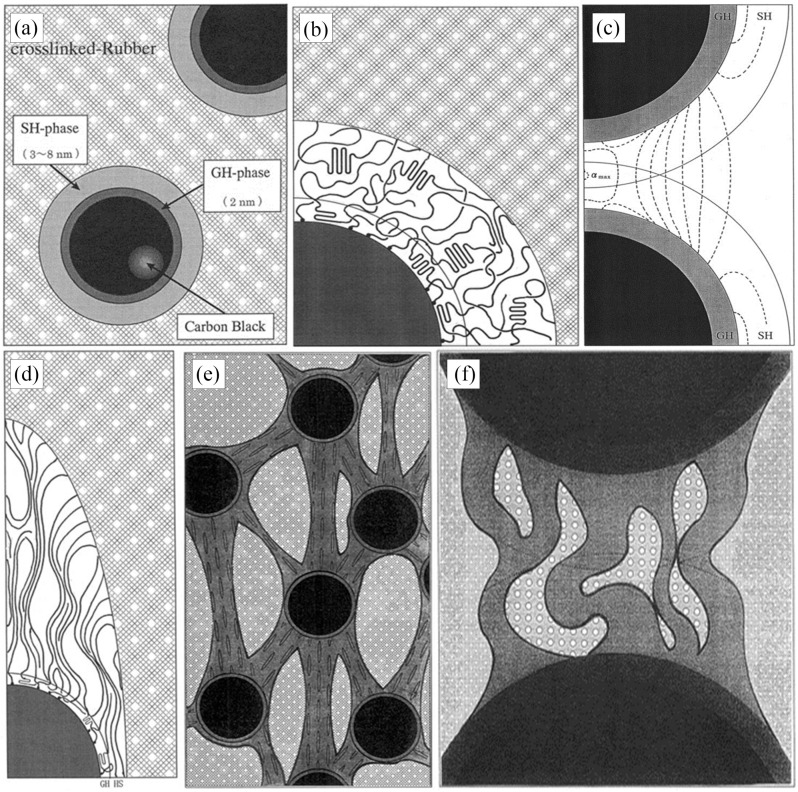
Diagrammatic representation of the binding gel bilayer model (**a**) A new double-layer interface model consisting of a GH layer and a SH layer. (**b**) Detailed molecular structures in the GH and SH layers as vulcanized. (**c**) Overlapped SH layers in triaxial expansion along extension direction at $\varepsilon_{0}=10\%$ and ϕ=0.2. (**d**) Molecular movements within the SH layer under large extension. (**e**) Supernetwork structure of carbon particles interconnected by strands of oriented molecules. (**f**) Buckling of extended and oriented molecular bundles [21].

An interfacial model based on the interaction of fillers and rubber macromolecules is called the binding rubber model. Based on this binding rubber model, Fukahori [21] suggested a new interfacial structure model based on the stress analysis results, accounting for the fact that the rubber’s volume expands in a large deformation state. As seen in Figure 4a,b, the bonded rubber bilayer structure model is the core of this model. According to the model, the structure of the binding rubber adsorbed at the carbon black’s periphery is made up of two parts: rubber layers with distinct moduli and an uncrosslinked structure. The inner structure is the glassy hard layer (GH) of the polymer in its glassy state, which has a thickness of around 2 nm, and the outer structure is the sticky hard layer (SH) of the polymer, which has a thickness of between 3 and 8 nm. The outermost layer, which has a thickness of 3–8 nm, is a sticky hard layer with limited polymer macromolecule mobility. The combined thickness of the two layers is around 5 nm for carbon black particles with a smooth surface and 10 nm for carbon black particles with a high degree of roughness on their outer layer.

The GH layer does not affect the sharp increase in stress under large magnitudes because the effect of the polymer glassy hard layer is limited to increasing the effective diameter of the carbon black particles, and its contribution to stress remains constant regardless of the amount of strain applied to it. While the GH layer cannot undergo orientation due to the poor mobility of the molecular chains, it does not contribute to the increase in the modulus of the rubber. At the same time, the viscous hard layer behaves similarly to the rubber matrix at small strains and contributes less to the modulus (Figure 4d). When the strain gradually increases, the SH layer undergoes orientation, a phenomenon that plays an important role in the increase in the rubber’s modulus. Furthermore, at higher carbon black contents, the SH layers separating various binding rubber particles may overlap, creating a super-network structure (Figure 4c). This super-network structure changes orientation and hardens under high strains, which adds to the modulus. Little holes will grow between the bundles of rubber macromolecular chains as they are assembled, and these holes can absorb some energy, postponing the rubber molecular chains’ eventual disintegration (Figure 4e). Simultaneously, the polymer’s volume expands under tension due to the existence of these microscopic pores.

Haghgoo et al. [22] investigated how multiscale fillers affected the electrical conductivity and resistivity of multiscale nanocomposites made of reinforced polymer and CB/CF. The outcomes demonstrated that the conductivity qualities and interoperability in the direction of electron flow were enhanced by the multiscale fillers. These enhancements were in good agreement with the microstructure’s multiscale filler modifications. Multiscale fillers can be added to polymer matrix composites’ microstructure to improve their electrical characteristics and reduce percolation thresholds.

Drawing on the micro-level analysis of the reinforcing model discussed above, we can employ several techniques to enhance the surface quality and surface activity of carbon black, resulting in NR composites with consistent filler dispersion. To create high-performance natural rubber composites, we can simultaneously lessen the force between carbon black particles, increase the force between carbon black and the NR matrix, and encourage the combination of carbon black and latex particles.

#### 3.1.2. CB/NR Composites Wet Mixing Preparation Process

Due to their hydrophobicity and/or high elemental carbon content (90~99%), carbon black particles agglomerate and produce unstable dispersions when dissolved in water at a pH close to the isoelectric point (IEP). Consequently, it is essential to regulate the surface charge of carbon black and limit its particle size to produce extremely stable carbon black aqueous dispersions [23]. An old issue in materials science is the creation of stable carbon black dispersions in water, either for use as pigments or as reinforcing filler particles in polymers. The application properties of carbon black are weakened due to their tendency to form agglomerates due to their tiny primary particle size and high inter-particle forces. Table 1 [24] illustrates the three primary categories into which the techniques employed in the literature to characterize the dispersion of carbon black can be separated.

As was previously noted, carbon black was dispersed in water using conventional surfactants or water-dispersible amphiphilic polymer architectures. Fresh natural latex was combined and agitated with a carbon black slurry that had been made by Alex et al. [25] while surfactants (alkali metal salts of fatty acids) were present. To create carbon black wet master gum, drying and acid flocculation were applied in the end. The results demonstrated that protein displacement and high adsorption onto the rubber particles were caused by adding fatty acid soap-based surfactants to the carbon black slurry. This process converts the protein-stabilized latex into a system that is stabilized by surfactants. The adsorbed anions combine with the acid in the surfactant enclosing the latex to generate undissociated surfactants, which rob the latex particles of their stabilizer. In addition, the latex’s Menni viscosity is low, and the surfactant might sensitize it to promote quick solidification.

Martínez-Pedrero et al. [26] studied the rheological properties of binary colloidal mixtures. During the sonication step, carbon black’s dispersion state was improved with the addition of sodium dodecyl sulfate (SDS). It was then agitated and combined with natural latex to produce a gel. The findings of the experiment demonstrated that the carbon black particles experienced a structural transformation in the binary colloidal mixes, bridging the natural rubber particles together. This phenomenon of bridging allows a generative network of fractal clusters to emerge, which in turn forms an elastic solid. The interparticle attraction energy between CB particles and NR droplets, which is impacted by the surfactant concentration, is primarily responsible for controlling this transition. The homogeneity of the dispersion is controlled before it loses stability by the adsorption of surfactant molecules on the carbon black surface, which modifies the interaction between the two particles. Applying shear can overcome the energy barrier preventing the bridging effect in the more stable scenario.

Using a similar strategy, Dong et al. [27] were able to effectively diminish the interaction between carbon blacks and improve the interaction between carbon blacks and rubber molecule chains, hence improving the dispersion of carbon blacks. Furthermore, it was shown that while the hardness and elongation at break showed the opposite pattern, the mechanical characteristics increased first and then dropped as the size of the carbon black particles increased. This is because more active sites are present in carbon black particles with smaller particle sizes since they have bigger specific surface areas. As a result, there is a greater degree of chemical bonding and physical adsorption between the rubber molecular chain and the surface of carbon black, resulting in improved bonding. The performance of vulcanized rubber made by the latex co-sinking process is still superior to that of conventional dry mixing, even though too-small carbon black particles are easy to agglomerate in the rubber matrix and reduce its mechanical qualities.

As previously shown, surfactants or polymers grafted with carbon black have improved dispersion stability. Carbon black can also employ its hydroxyl group and other active sites as low molecules for cationic, anionic, and free radical polymerization processes. The inter-particle resistive effect of the carbon black is enhanced by the grafting of the polymer onto its surface, which can successfully stop the carbon black from clumping together in the aqueous phase. The production of new or modified polymers has made extensive use of ultrasonic, high-energy electron beam (EB), and C-ray radiation in recent years as effective and ecologically acceptable free radical polymerization techniques.

By grafting acrylic acid (PAA) onto the CB surface with high-energy electron beam irradiation (EB), Jiang et al. [28] created water-dispersible CB. When compared to C-ray irradiation, EB is more efficient [29]. In addition to increasing the CB’s hydrophilicity and lowering its surface energy, grafting also improves the CB’s average aggregated particle size and dispersity when compared to unmodified CB. Fu et al. [30] grafted polyethylene glycol 400 onto the surface of carbon black using an in situ liquid phase grafting approach. Better dispersion of the modified carbon black in the rubber system increases the mechanical characteristics and the interaction between the modified filler and rubber. The dispersibility of carbon black can be effectively increased by the polymer graft modification method; however, because linear polymers are susceptible to molecular chain entanglement, carbon black particles may reaggregate and form new aggregates.

**Figure 5:**
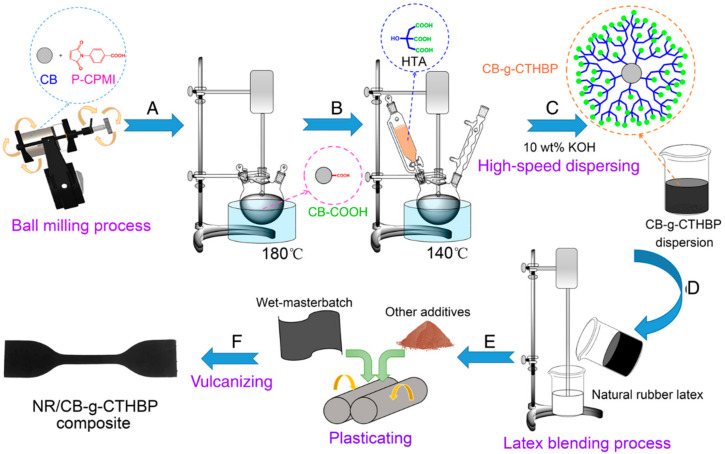
Schematic diagram illustrating the preparation route (**A**) Reaction to prepare CB-COOH. (**B**) Reaction preparation of CB-g-CTHBP. (**C**) Preparation of CB-g-CTHBP dispersions (**D**) Wet mixing of dispersion with latex. (**E**,**F**) Compounding to make rubber compounds [33].

Because of their distinct structure, high number of reactive functional groups, good solubility, low viscosity, non-entangled molecular chains, and simplicity of synthesis, hyperbranched polymers are of interest [31,32]. On the surface of carbon black, Han et al. [33] grafted end-carboxylated hyperbranched poly(2-hydroxypropane-1,2,3-tricarboxylic acid) (Figure 5). The modified CB greatly enhanced the dispersibility and wettability of the composite; its average particle size was significantly smaller, and its particle size distribution was narrower, according to the results. This could be explained by the hyperbranched polymer development on the CB surface, which raises the spatial site resistance and electrostatic repulsion between CB particles. This, in turn, increases the repulsive force between CB particles, making it more challenging for CB to assemble [34,35]. A significant amount of carboxyl groups, which are hydrophilic oxygen-containing functional groups, are present in the topmost layer of the hyperbranched polymer layer and may help to improve the dispersion of carbon black in water.

As was previously indicated, the emulsion coalescence process can be used to create composites by adding coating resin to a mixed system of carbon black and latex to form a powder system [36]. With the use of the emulsifier AEO-9, Lin et al. [37] generated the CB emulsion, which they subsequently mixed and combined with NR latex to create a powder system. After being heated in a water bath, carbon black and natural rubber thermally aggregate. Afterward, flocculation and drying were accomplished using a CaCl_2_ solution. According to the findings, natural rubber composites filled with carbon black and powdered had superior mechanical and dynamic properties than those made by traditional dry blending. The improved composite properties were also attributed to the good dispersion of CB in the rubber matrix and the improvement in rubber–filler interactions.

Process approaches can also be used to obtain a homogenous mixing of carbon black with natural latex in addition to carbon black modification. The earliest method to homogeneously disperse carbon black in latex was developed by Cabot [38,39]. It involved using high-pressure jetting technology to quickly mix and flocculate natural latex with carbon black slurry, but the equipment and process requirements are complex. Afterward, Han et al. [40] used high-speed impact jet processing to directly combine natural rubber latex (NRL) with carbon black (CB) to create rubber composites. The filler dispersion and latex were combined using a side-mounted jet mixer to create a combination that was then baked in an oven to create master rubber. The findings demonstrated that, in comparison to the traditional dry procedure, the jet compounding technique allowed for a more uniform dispersion of CB into natural rubber. Furthermore, a comparison of the jet Reynolds number mixing forms revealed that the carbon black was more likely to agglomerate and have bigger particle sizes in the matrix and that the particles in laminar flow mixing were subjected to less shear stress. Turbulent jet mixing, on the other hand, successfully prevents particle agglomeration. It improves the interaction between rubber and fillers, diminishes the network of fillers, and improves filler dispersion in the rubber matrix, all of which increase load transfer efficiency.

**Figure 6:**
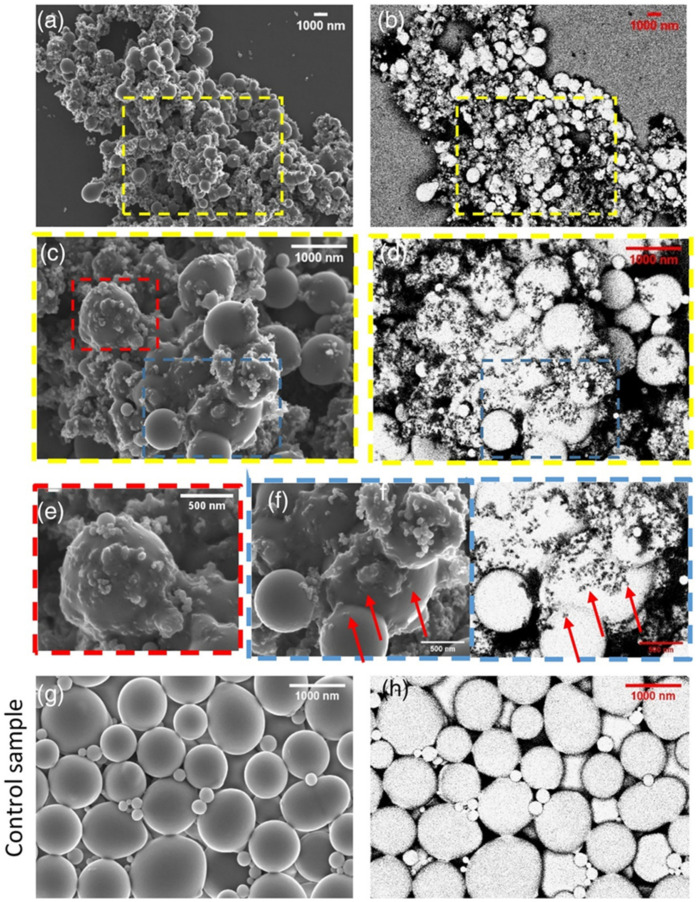
FESEM micrographs of the NR–CB heterocoagulum obtained with sonication during mixing: Mass ratio in the liquid suspension = 40% CB/rubber and the structure was fixed at t = 1 h. (**a**) Large view SE and (**b**) BSE images of the same region showing the homogeneous distribution of NRs and CB filler (magnification: ×5000). (**c**) Magnified SE and (**d**) BSE views corresponding to the yellow squared region in (**a**,**b**) (magnification: ×10,000), highlighting the partial coalescence between the NR globules that are surrounded by several small CB aggregates. (**e**) Magnified SE of the red boxed region in (**c**) (magnification: ×80,000). (**f**) Magnified SE and BSE views of the blue boxed region shown in (**c**,**b**) (magnification: ×40,000), showing that the CB filler partially sinks toward the inner part of the NR globules. (**g**) Magnified SE and (**h**) BSE views corresponding to the control sample composed only of sonicated NR globules [42].

Using ultrasonic technology to pretreat natural latex can boost the reinforcing effect by increasing the contact area between rubber hydrocarbon particles and filler particles and rupturing the protective barrier. Simultaneously, filler particle size can be decreased via ultrasonic treatment, improving filler dispersion uniformity. Galinovskiy et al. [41] explored the effect of ultrasound and ultrasonic jet treatment methods on the dispersion of nanosuspensions. The experimental results indicate that ultrasonic jet dispersion is a potential method for particle deagglomeration and a solution to the issue of achieving the required degree of dispersion in suspensions. Using a novel technique called field emission scanning electron microscopy (FESEM), Cattinari et al. [42] examined how the nanoscale structure of NR–CB concretions changed as the solvent evaporated. Osmium vapor was used to specifically chemically immobilize the sample, preventing it from submerging in the immobilizer-containing liquid solution while maintaining the colloidal structure of the NR particles. We looked into and examined the impact of external physical stressors (shear and ultrasonic) on the coagulation of NR latex and CB slurry. It was discovered that combining the two parts in a colloidal suspension while subjected to sonication kept the CB filler in the form of tiny aggregates (20–200 nm), with excellent filler homogeneity surrounding the NR pellets (Figure 6). It was determined through experimentation that the NR’s sonication did not affect the structure (Figure 6g,h). The NR and CB were evenly distributed, and heterogeneity was mostly responsible for the clots’ aggregation (Figure 6a,b). Some of the aggregates between the NR are highlighted in images captured at high magnification (Figure 6c,d). Some of the aggregates have sunk into the sphere, as shown by the red arrows in Figure 6f, and these aggregates formed a distinct contact on the sphere’s surface (Figure 6e).

Using the grinding balls’ inherent gravity and the impact of the balls colliding, ball milling technology breaks up aggregates and helps refine particle size. Yamamoto et al. [43] used a ball mill under various ball milling conditions to study the dispersion characteristics of carbon black in solvents. The results of the trials demonstrated that the dispersion performance rose as the stirrer speed and bead loading ratio increased and that there was a strong correlation between the two parameters and the impact energy of the beads. To prepare silica and carbon black dispersions for the creation of natural rubber composites, Hamran et al. [44] used ball milling sonication. The dispersion treated with ultrasonication followed by ball milling demonstrated the best dispersing ability, and the results indicated that this combination of treatment methods was the optimum way to minimize the particle size of silica and carbon black.

Sui et al. [45] suggested a completely formulated wet mixing approach for carbon black. Using all of the formulation’s fillers, they ball-milled the pretreatment to create an aqueous dispersion slurry, which was then combined physically, via stirring, with the latex. Ultimately, a twin-screw was used to accomplish the flocculation and dewatering steps, and an oven was used to dry and further treat the rubber masterbatch. The generated rubber composites containing carbon black exhibited a homogeneous dispersion and distribution, tiny particle size, and no visible aggregation phenomenon due to the completely formulated wet continuous mixing procedure that combines wet and continuous mixing methods. The carbon black dispersion was enhanced in comparison to the conventional dry mixing, and the composites’ tensile strength, elongation at break, and cut resistance all increased by 9.4%, 9.6%, and 35%, respectively. On the other hand, constant wet mixing permits the molecular chains in natural rubber to be evenly distributed throughout the rubber by minimizing chain breaking. Consequently, the fully formulated wet blend’s Menni viscosity is higher than the dry blend’s.

In summary, dispersant or surfactant can be used to modify carbon black, even though it is dense, hydrophobic, and settles a lot in aqueous dispersion. Furthermore, the modified carbon black frequently cannot match the latex’s settling speed due to its differing polarity and density, which may have an impact on the carbon black filler’s stability and dispersion in the rubber matrix. Since the rubber phase in the mixed product is not cross-linked and tends to aggregate due to the thermodynamics of carbon black, we should try to take steps to dehydrate and dry the product quickly to reduce its viscosity and lengthen its survival period. To minimize wastewater, waste gas, and solid waste, shorten the production cycle, reduce the three wastes, and improve product quality and performance, wet mixing technology’s benefits for the economy and environment should also be carefully taken into account during the production process. This is because there is an antagonistic relationship between the search for wet mixing technology’s advantages and the development of low-carbon, energy-saving, and environmentally friendly directions.

### 3.2. NR/Silica (SiO_2_) Composites

#### 3.2.1. Mechanism of Silica Reinforced Rubber

Silica’s reinforcing process is quite similar to that of carbon black. When compared to carbon black, silica’s biggest distinguishing attribute is its surface activity. Silica is an amorphous structure of silicon dioxide, having silicon atoms in the center and oxygen atoms at the apex, forming an irregular tetrahedral shape. The silicon atoms on the surface are unevenly organized, and the hydroxyl groups that connect them change depending on the chemical reaction. The numerous hydroxyl groups on the surface of silica can be classified into three types: double hydroxyl, isolated hydroxyl, and neighboring hydroxyl.

The adsorption–slip theory, which states that fillers strengthen rubber primarily through molecular chain slip when the composite is stretched by external pressures, is now generally accepted. The addition of silica filler to rubber results in the binding of several silica particles to the rubber molecular chain, which in turn binds each silica particle to numerous rubber molecular chains. When an external force is applied, the rubber molecules will align themselves in the force’s direction. This is because the silica particles are separated by several rubber molecular chains, each of which has a different length. The longer chains are forced to slip on the silica surface when the shorter chain segments restrict the orientation of the larger chains after they have finished their tensile orientation. The molecular chains connecting the silica particles are completely stretched as the orientation proceeds. To achieve a greater modulus, each rubber molecular chain segment is currently subjected to a more uniform external force thanks to the silica-reinforcing effect of the rubber. This reinforcing structure will be destroyed, the slip orientation will cease, and the composite material will fracture when the external force exceeds a particular critical amount.

The silica exhibits a high specific surface area and strong surface adsorption. It is typically presented in an aggregated state, which allows for a larger contact area with rubber and promotes the formation of a bond between the two materials. The crystallization effect of the particles causes the adsorption layer to rise, resulting in a smaller particle diameter than the spacing between them, which plays the role of physical reinforcing [46]. Chemical reinforcement can be created when the double bond of the rubber molecular chain reacts with the hydroxyl group present on the surface of silica [47]. Bonded rubber is created when the alkaline reactive groups of rubber react with the acidic hydroxyl groups on the surface of silica [48]. This reaction between the two groups creates silica-reinforced rubber.

#### 3.2.2. SiO_2_/NR Composites Wet Mixing Preparation Process

Silica (SiO_2_) is the preferred filler for tire tread applications because it has lower rolling resistance and higher wet traction than carbon black. Utilizing silica as much as possible instead of traditional carbon black while preparing NR-based green tire rubber compounds is crucial for the automotive sector, given the current state of the world and environmental concerns [49]. Generally speaking, rubber’s mechanical qualities are enhanced by silica addition. However, silica is more polar due to the abundance of hydroxyl groups on its surface, which reduces the interaction between silica and rubber. Furthermore, silica dispersion is typically poor in carbon black-filled compounds made using the traditional mixing method. The viscosity rises dramatically with substantial additions of silica, which complicates processing and puts undue strain on processing machinery. The strong contact between silica particles is responsible for the viscosity increase. Two approaches can be taken to address this issue: either the hydrophilic silica surface is modified, or the rubber’s non-polar structure is functionalized to make it more polar [50]. Table 2 and Table 3 include a list of papers on the wet filling of natural latex with silica.

Silane coupling agents (SCAs) are used to alter the silica surface. Amphoteric surface modifiers and interfacial compatibilizers are silane coupling agents (SCA). Chemically speaking, SCA has at least one alkoxy group that, by dehydration condensation, can form stable Si-O-Si structures with SiO_2_ particles. Reactive groups, particularly reactive sulfur atoms, are also present in SCA and can react chemically with diene rubber macromolecules.

The most practical and technologically advantageous silane coupling agents for silicone-based NR compounds are sulfide–alkoxy silane coupling agents, according to research by Kaewsakul et al. [62] on silane coupling agents with various particular functionalities. By comparatively offering higher filler–rubber contacts than single alkoxy or sulfide-based silanes, these chemicals can effectively lower the viscosity and filler–filler interactions of the compounds, leading to a notable increase in mechanical characteristics.

By altering SiO_2_ with 3-mercaptopropylethoxybis(tridecylpentamethoxy)silane (Si-747), a long-arm silane coupling agent, Zheng et al. [63] created NR/SiO_2_ composites. Si747 is a type of SCA with two long arms that can form abundant and concentrated hydrogen bonds with SiO_2_, and the creation of an NR/NR masterbatch will be more satisfying when the total amount of Si747 is sufficient to reduce the electronegativity of SiO_2_. When the total concentration of Si747 is sufficient to counteract the electronegativity of SiO_2_, a more suitable silica/NR masterbatch will be prepared. Some papers on wet filling of natural latex with silica are listed in Table 2 and Table 3.

The hydroxyl groups on the surface of silica react with conventional silane coupling agents to produce a lot of volatile organic compounds (VOCs), which degrade rubber composite performance and contaminate the environment [64]. By reacting fatty alcohol polyoxyethylene ether (AEO-X) with varying polyether lengths with bis[γ-(triethoxysilyl)propyl]-tetrasulphide TESPT (Si69), Li et al. [65] created a range of novel coupling agents with low VOC emission, which they then integrated into silica/natural rubber nanocomposites. The findings demonstrated that the new coupling agents could properly balance the tires’ “magic triangle” performance and greatly enhance silica dispersion in the rubber matrix. SiO_2_/NR composites showed improvements in wet slip resistance of 14.2%, abrasion resistance of 20.3%, and rolling resistance of 15.8%.

**Figure 7:**
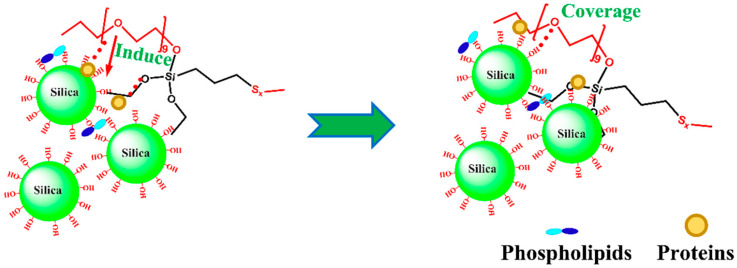
Schematic diagram of silica modified with Mx-Si69 induced by NRCs [66].

A set of low volatile organic compound (VOC) Mx-Si69 couplers (Figure 7) was applied to SiO_2_/NR nanocomposites by Zhai et al. [66] These couplers correspond to the number of ethoxyl groups in bis-(γtriethoxysilylpropyl)-tetrasulphide (Si69) substituted by aliphatic polyether chains (x = 1, 2, 3, 4, 5, 6). Excellent performing tire treads were ready. First off, M1-Si69 is the optimal option for both Si69 and Mx-Si69. In addition to giving the tires exceptionally good anti-slip performance and very low energy loss, applying the recently developed M1-Si69 coupling agent to SiO_2_/NR nanocomposites can also drastically cut down on VOC gas emissions. 

Different ratios of TWEEN-20 and TESPT were chosen by Xiao et al. [67] to alter silica and create NR nanocomposites. The lengthy fatty chain portion of TWEEN—20 is more suited for rubber, while the terminal hydroxyl group and polyether portion can interact chemically and physically with silica. Consequently, TWEEN-20 can significantly improve silica dispersion in NR composites. In addition to lowering volatile organic compounds (VOCs), the synergistic interaction of TWEEN—20 and TESPT can enhance the ideal dispersion of silica in the rubber matrix. When the ratio of TESPT-to-TWEEN-20 was 2:1, the rubber nanocomposites’ dynamic and static characteristics were at their best.

Premature scorching during the blending process is a problem caused by TESPT because the silanization reaction demands a temperature range of 140 °C to 150 °C. Alternative silanes such as octanoylthiopropyltriethoxysilane and bis-triethoxysilylpropyl disulfide (TESPD) were introduced to get around this problem. Therefore, the effectiveness of silica-filled rubber products mostly depends on the proper arrangement of essential system components [68]. When Kaewsakul et al. [68] examined two silane coupling agents, TESPT produced better mechanical qualities, filler–rubber interaction, and dispersion stability. TESPD extended the rubber’s coking period, with ideal loadings of roughly 9.0 weight percent (about silica) for both. Additionally, it was shown that DPG, when used as a catalyst, may enhance the silanization reaction between the silane coupling agent and silica, with the best loading being 2.0 weight percent.

To improve the interaction of silica-filled compounds, certain polar rubbers with functional groups that can interact with the silica surface have been studied as silane coupling agent alternatives. Using epoxidized natural rubber (ENR) as a bulking agent, Sengloyluan et al. [69] studied tire tread rubber made of silica-based natural rubber (NR). Epoxidized natural rubber was added, which enhanced the dispersion of silica in the rubber and decreased the rubber’s Menni viscosity, Payne effect, flocculation rate constant, and packing network factor. Epoxidized natural rubber (ENR) was employed by Xu et al. [70] as an interfacial modifier to enhance the mechanical properties of SiO_2_/NR composites. This resulted in the formation of covalent bonds between the epoxy groups on the ENR chain and the Si-OH groups on the surface of silica, which enhanced the dispersion of SiO_2_ in the rubber matrix and reinforced the interfacial bond between NR and SiO_2_. Not only does the ring opening response happen during vulcanization, but it also happens during compounding, and the latter is more significant because of the interaction of temperature and mechanical forces. He et al. [71] prepared a silica dispersion by a high shear process using liquid natural rubber (NRL) as a bulking agent and then blended it at low speed with latex. Finally, flocculation and acetic acid drying were used to create natural rubber–silica composites. The tensile and tear strengths increased by 37.9% and 18.9%, respectively, and the silica dispersion in the NR matrix was enhanced with the addition of NRL, which also encouraged vulcanization. Natural rubber masterbatch was created by Wang et al. [72] from NRL-treated silica. It had improved wet slip resistance, good compatibility with rubber, good dispersion in rubber matrix, and a diminished Payne effect of the composites. The performance of the rubber composites was significantly enhanced by the dispersion and interfacial adhesion of silica in the rubber matrix.

The most practical method for silica surface functionalization is to use silica silanol groups that have reacted through the sol-gel reaction with appropriate organoalkoxysilane reagents. However, because of spatial site resistance effects, the conversion of organosilanes to SiO_2_ particles via the sol-gel process somewhat reduces as the size of the substituent groups on the organosilanes rises. To increase the compatibility of organosilanes with NR, Theppradit et al. [51] modified the SiO_2_ surface silica hydroxyl groups with methyl, vinyl, and aminopropyl groups using MTES, VTES, and APTMS, respectively. This resulted in the modified SiO_2_ particles having distinct surface properties depending on their hydrophobicity. The vulcanization features, as well as the physico-mechanical, thermal, and dynamic mechanical properties of the filled NR vulcanized rubber, were enhanced when compared to the unmodified SiO_2_ particles due to a greater interfacial connection between the modified SiO_2_ particles and the NR matrix.

To create NR composites, Jansomboon et al. [52] blended SiO_2_/GE blends made using the hybrid approach with NR by latex blending. The products were then cured with EB radiation. The outcomes demonstrated that NR composites might be crosslinked using EB irradiation to enhance their tensile characteristics. The NR composites filled with 2.5 parts SiO_2_/0.056% GE blend had the best tensile strength at an EB irradiation dose of 150 kGy. Additionally, the modulus and tear strength were significantly enhanced by irradiation.

**Figure 8:**
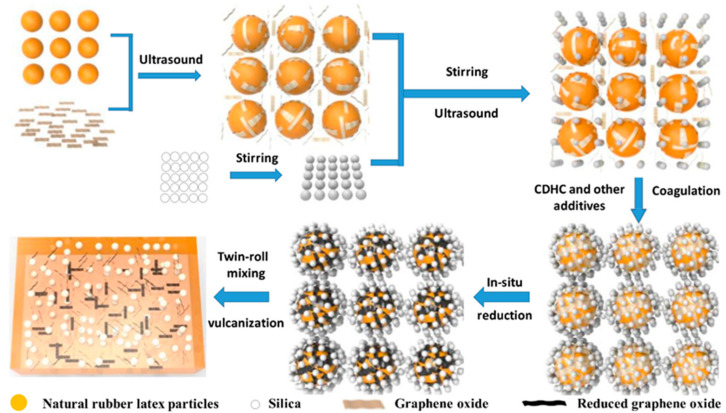
A wet compounding process combined with ultrasonically assisted latex mixing (WCL) method for the preparation of reduced graphene oxide (rGO)/silica/natural rubber (NR) composites [54].

These days, a lot of fresh additives are also applied to enhance the silica-based reinforcing of natural rubber. The impact of low eutectic solvent (DES) modified silica on the characteristics of natural rubber composites was examined by Yang et al. [53] Choline chloride and urea were combined in a 1:2 molar ratio to create DES. By forming hydrogen bonds with silica, the low eutectic solvent can enhance the compatibility of rubber and silica. The tensile strength, grip strength, crosslink density, and constant elongation stress of the composites were enhanced when the amount of DES was three parts. Additionally, the rolling resistance of vulcanized rubber was reduced while maintaining good wet slip resistance. Wang et al. [54] developed graphene oxide (rGO)/SiO_2_/natural rubber (NR) composites by combining wet mixing with ultrasound-assisted latex mixing (as shown in Figure 8). Bladder cysteamine dihydrochloride (CDHC), a new interfacial modifier with coagulation capabilities, was added to the procedure; the latex particles, SiO_2_, and CDHC’s electrostatic interactions served as a coagulant. As a coagulant during preparation and an interfacial compatibilizer in the final composites, CDHC can establish potent electrostatic contacts with rGO, silica, and NR latex particles.

Xia et al. [73] used L-lysine as a catalyst in the hydrolytic condensation reaction of ethyl orthosilicate to create nanoscale autonomous monodisperse silica (AS) particles. Next, using the latex blending process, NR latex was combined with AS dispersion to create a silica/natural rubber (NR) masterbatch. The AS/NR composites demonstrated superior tensile strength, abrasion resistance, and resilience in comparison to PS/NR due to the presence of more bound rubber and a decrease in filler–filler interactions, as demonstrated by the use of commercially available precipitated silica (PS) as a reference.

**Figure 9:**
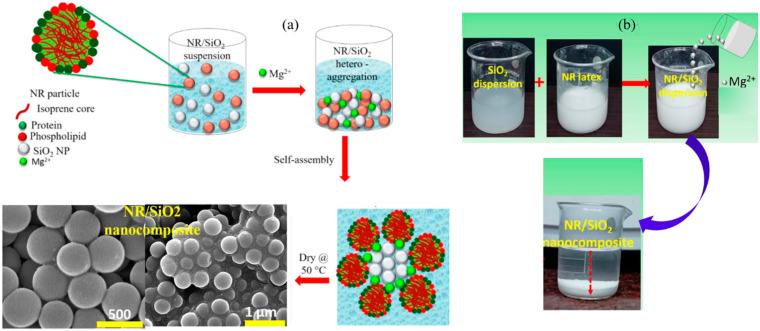
(**a**) Schematic of the synthetic pathway for the formation of 3D self-assembled NR-SiO_2_ nanocomposite and its SEM images. (**b**) Depiction of nanoscopic aggregation in NR-SiO_2_ nanocomposite in the presence of 100 mM Mg^2+^ [55].

Hydrogen bonding, covalent bonding, hydrophobic contacts, ligand bonding, electrostatic attraction, and charge-transfer interactions are the common forces used in the self-assembly process, which may also be used to synthesize NR/SiO_2_. A novel technique was developed by Curk et al. [74] to produce 3D self-assembled SiO_2_-NR nanocomposites in the presence of Mg^2+^ and in aqueous phase conditions. Negatively charged NR and SiO_2_ colloidal particles can be positioned on an advantageous NR-SiO_2_ heterogeneous condensation by creating molecular bridges. Sattar et al. [55] used self-assembly and latex compounding procedures to manufacture SiO_2_/NR nanocomposites (Figure 9). Ultrasonic dispersion was used to create the suspensions, which were subsequently combined with latex, acid flocculated, and dried. Moreover, emulsion blending and Mg^2+^ particle-induced heterogeneous coalescence self-assembly were utilized to achieve the homogeneous dispersion of SiO_2_ particles in the NR matrix. In contrast to NR-NR or SiO_2_ NP-SiO_2_ NP self-aggregation, divalent metal ions were crucial in creating molecular bridges between negatively charged stable NR and SiO_2_ colloidal particles under aqueous-phase conditions. It was shown that SiO_2_ NP-SiO_2_ NP self-aggregation or NR-NR hetero-aggregation were preferable.

Using liquid mixing of model NR latex–silica NPs, Cattinari et al. [56] employed nanofluorescence imaging to thoroughly analyze the structure and evolution of heterogeneous aggregates generated by colloidal instability. SiO_2_NPs were found surrounding the NR spherical particles as a result of Mg^2+^ ion-mediated electrolyte-responsive contacts between NR and filler particles; however, this interaction did not cause a film-forming mechanism but rather stopped the aggregation between the spheres. Additionally, throughout the interaction, the distribution of proteins and lipids within the biofilm does not alter, which restricts the biofilm’s ability to shift to a membrane state. Either the solvent evaporating or the liquid suspension being sheared can accomplish this change. Shearing has the advantage of quickening the complex’s formation by accelerating the kinetics of heterogeneous aggregation and facilitating the polymerization between NR spheres. The composites had a network of elliptical ring-like structures grouped in a regular pattern that mimicked the original NR spherical appearance. As a result, the liquid state interactions established by NR are directly related to the dispersion of SiO_2_NPs in the composites.

By mechanically combining silica solutions, blending them with NR latex, and then drying and agitating the mixture, Ryu et al. [57] created SiO_2_/NR composites. Without compromising other rubber qualities, the wet masterbatch rubber’s high silica dispersion, low Payne effect, and low loss modulus prevent silica aggregation and result in low viscosity, good starting safety, and low rolling resistance. Kim et al. [75] created silica slurry by stirring, flocculation by mixing with the latex plus aqueous acetic acid solution, and drying by mixing. They also prepared ESBR/SiO_2_, BR/SiO_2_, and NR/SiO_2_ composites by wet and dry techniques, respectively. There was an 82% improvement in the dispersion of silica in the rubber matrix. The wet masterbatch showed good elongation, tensile strength, and abrasion resistance, along with low Menni viscosity and soft filler flocculation because of the silica’s good dispersion.

NR/silica and NR/ENR/silica composites were made using the wet masterbatch method by Wang [58]. The silica suspensions were made by ultrasonic dispersion, blended with ENR and NR latex, and then solidified by adding ethanol. Wet masterbatch-based composites demonstrated comparable wet slip resistance with reduced rolling resistance, an important characteristic of materials used in high-performance green tire treads.

**Figure 10:**
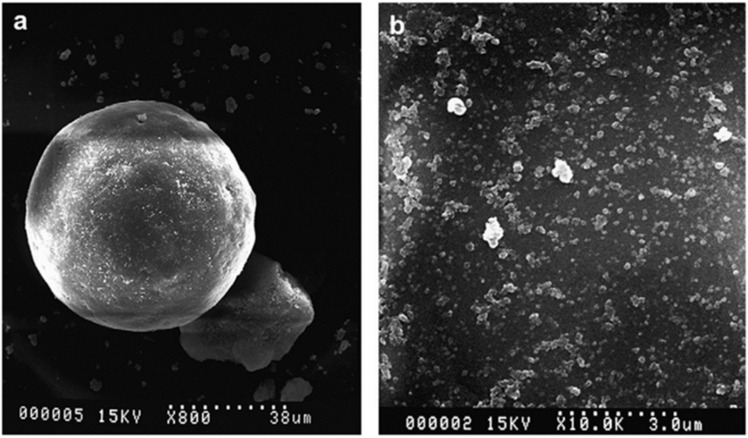
SEM micrographs of silica suspension; (**a**) before grinding and (**b**) after grinding [61].

After processing the silica with a ball mill, Prasertsri et al. [59] created well-dispersed silica suspensions, as shown in Figure 10. They also created a silica/NR masterbatch by flocculating it with calcium chloride after blending it with natural latex. Because the moisture during the preparation process protected the curing adsorption on the silica surface, the wet preparation of silica/NR masterbatch did not experience the phenomenon of delayed vulcanization that is associated with conventional silica-filled rubber. This is in contrast to the conventional preparation method. Furthermore, the wet-preparation blend has a stronger reinforcing effect than the conventional dry blend when Si69 is absent. Subsequently, utilizing a latex system and fumed silica (FSi) and precipitated silica (PSi) suspensions as raw materials, Prasertsri et al. [60] developed a silica/natural rubber (NR) masterbatch. The findings demonstrated that while a stirred ball mill and an ultrasonic water bath could both produce well-dispersed FSi suspensions, only the latter could produce well-dispersed PSi suspensions. The loss of silica in the composite FSi/NR masterbatch was significantly lower than in the dry preparation of silica-filled NR, and the silica content of the FSi and PSi-filled samples gradually increased the composite viscosity, cross-linking initiation point, and stiffness. Phumnok et al. [61] used a response surface approach in their ball milling process to prepare silica dispersions. When the silica content was around 22% and the ball milling time was 62 h, the best dispersion of silica suspension was achieved. The wet method offers a longer coking and curing time than the dry method, which is beneficial for process control. The silica particle size was reduced to 4.9 mm. This is especially true at high silica loading. The rolling resistance is reduced and the mechanical qualities are superior to the dry process, making them appropriate for the manufacture of green tires. Neena et al. [76] examined how the processing technique affected the creation of SiO_2_/NR composites and demonstrated that the technique had a significant impact on how fillers are distributed throughout the polymer matrix. A nano-silica network encircled the rubber microspheres, created by the NR/nano-silica composites made using the latex stage mixing-cast film forming technique. The enhanced crosslink density and solvent resistance of the nanocomposites suggest that the rubber–filler interactions are functioning well.

Nano-SiO_2_/NR composites were made by Lin et al. [77] via in situ production and coalescence. By adding Si69, the vulcanized rubber’s performance was further enhanced. It offered reduced rolling resistance and increased wet resistance when compared to the vulcanized rubber without Si69. The mechanical and dynamic qualities outperformed those of SiO_2_/natural rubber composites made using the traditional dry mixing technique.

A unique experimental technique based on sol-gel chemistry was proposed by Utrera-Barrios et al. [78] for the in situ synthesis of Si and Zi nanoparticles in previously reinforced nano-reinforced materials. The outcomes demonstrated that the Si particles produced in situ were reinforcing at low to medium strains (500%) and raised the rubber’s stiffness because of their improved hardness and resilience to abrasion.

A novel technique called atomization drying allows for quick flocculation drying without the need for a flocculant. The latex mixture can be atomized to create tiny droplets with a high specific surface area. The high temperature can then be used to evaporate the moisture from the droplets quickly, allowing for drying. To create natural latex/silica composites, Bian et al. [79] combined silica and latex, sprayed it via atomization with a spray cannon onto a self-designed high-temperature circular roller for quick drying, and then scraped it off with a scraper. The composites made by atomization combined with the high-temperature sputtering and drying (A-HTS) method had the best physical and mechanical properties when compared to the strong acid flocculation method, the natural drying method, and the general mixing method of cigarette flake rubber. In the dynamic mechanical property analysis, these composites also demonstrated excellent rolling resistance and anti-slippery properties. A-HTS is able to ensure good dispersion and uniform distribution of fillers in the latex by preventing filler agglomeration after dispersion and promoting uniform dispersion. Graphene oxide/silica/natural latex composites were made by Wang et al. [80] using traditional drying methods, mechanical stirring, dry ice expansion pre-dispersion, and spray sputtering drying, respectively. The spray sputtering method produced greater silica dispersion in the rubber matrix and higher physico-mechanical characteristics when compared to the other three procedures. When compared to the dry approach, the composites made using the spray sputtering drying process had better tensile strength, tear strength, and abrasion resistance by 27.2%, 43.6%, and 24.6%, respectively. SiO_2_/NR composites were made by Zhu et al. [81] using a continuous wet mixing method that included twin-screw mixing, high-temperature flash drying, and ball milling to treat the filler dispersion. The continuous wet mixing approach facilitated silica dispersion and distribution throughout the preparation process, in contrast to traditional dry mixing. The rubber materials showed improvements in tensile strength, abrasion resistance, and fracture energy index of 20.4%, 45.1%, and 14.3%, respectively.

**Figure 11:**
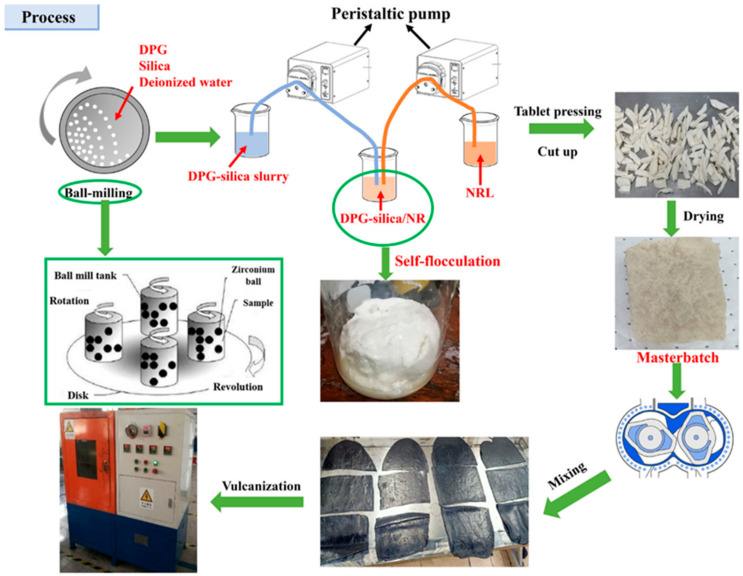
Silicon dioxide-loaded 1,3 diphenylguanidine (DPG) combined with self-flocculation technology process [82].

As research progressed, scientists started examining how silica and natural latex self-flocculate to prevent flocculants from altering the characteristics of rubber materials. The promoter 1,3 diphenylguanidine (DPG), which has pro-sulfurization and pro-silanization properties, was loaded onto the green filler silica by Xiao et al. [82] using high-energy ball milling technology, as shown in Figure 11. Simultaneously, DPG destroyed the latex’s ionization equilibrium and accomplished self-flocculation.By promoting the dispersion and silanization reaction of silica, the DPG-induced self-flocculation approach helped to produce a more comprehensive filler–rubber network and three-dimensional crosslinked network. The ball milling–autoflocculation technology raised the silanization index, tensile strength, and aging factor by 8%, 31%, and 19%, respectively, in comparison to the conventional dry mixing approach. Rolling resistance is reduced, and the pace of cure is accelerated.

By using a wet mixing methodology instead of the conventional dry mixing method, SiO_2_/NR composites can be prepared with better silica dispersibility, filling capacity, and reinforcing capacity. Processing is made extremely difficult by the wet masterbatch’s high Menni viscosity and lack of mixing. To summarize, silica can be modified through surfactant treatment, which can help to partially restore the masterbatch’s Mooney. Therefore, it is anticipated that wet blending of silica will have a big development space if the issue of wet masterbatch processing difficulties is successfully resolved.

### 3.3. NR/Carbon Nanotubes (CNTs) Composites

#### 3.3.1. Mechanism of Carbon Nanotubes Reinforced Rubber

Carbon nanotubes are formed of one or more layers of graphite flakes twisted at a specific helix angle. They are seamless tubular materials with diameters that are nanometers. Consequently, based on the various layers of graphite flakes, carbon nanotubes can be divided into two groups: single-walled carbon nanotubes (SWCNTs) and multi-walled carbon nanotubes (MWCNTs).

When Nah et al. [83] compared the CNT-filled rubber composites’ reinforcement mechanism to that of CB, they found that, for the same filler quantity, the mechanical properties of CNTs/NR composites were much better than those of CB/NR composites. Conversely, more stress softening at both low and high strains and volume expansion in tension were linked to the reduced amount of bonded rubber. These findings imply that there is not much interfacial contact between rubber and CNTs and that interfacial connections are easily broken when the materials are stretched. Therefore, rather than strong interfacial adhesion, the most significant element for the enhancement of CNTs appears to be their high aspect ratio, which causes physical entanglement and efficient load transfer.

The uniaxial tensile big deformation behavior of MWNT/NR nanocomposites with varying MWNT volume fractions was studied by Deng [84]. In the tensile strain range of 150% to 400%, the materials exhibited a highly nonlinear stress–strain relationship. To comprehend the possible enhancement process under massive deformation, a theoretical model of MWNT/NR composites was created. The model indicates that the macroscopic mechanical response is significantly influenced by the orientation evolution and persistence length of MWNTs.

**Figure 12:**
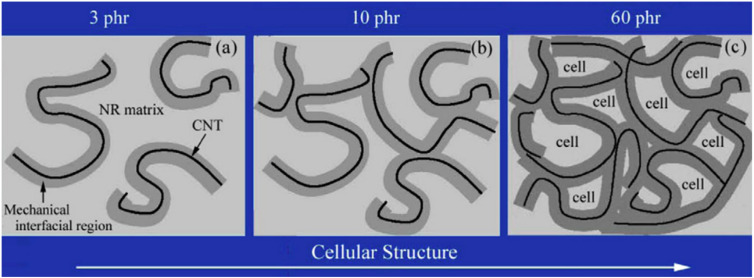
Schematic diagram of the cellulation model formed in CNTs/elastomer composites. (**a**) Percolation, (**b**) partial cellular structure, and (**c**) three-dimensional cellular structure [85].

A model for the “cellular structure” that forms in CNT/elastomer composites was proposed by Wang [85] (Figure 12). The mechanical interfacial zones of the CNT and the CNT itself are consistently and separately distributed in the NR matrix at low CNT loadings (Figure 12a). This composite has relatively low mechanical characteristics since there is no contact between the CNTs. The CNTs and the mechanical interface regions are tightly entwined into a network to generate a three-dimensional structure as the CNT loading increases. As a result, the mechanical interface area and the CNT itself make up the tiny unit that is known as the NR matrix. “Cytoarchitecture” refers to the three-dimensional linkages that exist between the CNT and the mechano-interfacial area. The “cell” is restricted to the NR matrix, and the mechanical interfacial zone functions as a cell wall. CNT/NR composites are essentially collections of cells. There is the formation of a cellular structure at high CNT loading. A portion of the cell-like arrangement developed when the filling amount of CNTs reached 10 phr, as seen in Figure 12b. A significant number of vesicles developed and dispersed throughout the matrix when the addition of CNTs reached 60 phr (Figure 12c). The significant improvement in mechanical characteristics can be attributed to the creation of the cell-like organization.

To predict the creep behavior of CNT–polymer nanocomposites, Hassanzadeh-Aghdam et al. [86] proposed a new modeling approach based on micromechanics. They discovered that agglomeration of CNTs affects and reduces the creep resistance of CNT–polymer nanocomposites and that important factors affecting the composites include random dispersion, transverse anisotropy, and viscoelastic interfaces of CNTs.

Because of their exceptional qualities, which include high mechanical, high thermal, and high electrical conductivity, carbon nanotubes (CNTs) have garnered a lot of interest [87,88,89]. In the rubber business, adding carbon nanotubes (CNTs) to rubber products can enhance their mechanical, electrical, and thermal conductivity even further. Additionally, achieving the optimal reinforcing effect and maximizing the exceptional performance of rubber requires a good dispersion of CNTs. As a result, the rubber and CNT mixing procedure is essential. CNTs are quickly entangled with each other due to their wide aspect ratio and the strong van der Waals forces between two tubes [90,91], which makes it challenging for them to be evenly distributed throughout the matrix. Liquid-phase mixing has been demonstrated to further increase the dispersion of carbon nanotubes in the rubber matrix and boost their reinforcing capacity [92,93].

#### 3.3.2. CNTs/NR Composites Wet Mixing Preparation Process

Many surface modification techniques (as shown in Table 4), based on mechanical milling, covalent bonding of functional groups, noncovalent encapsulation, or adsorption of medium molecules on the CNT surface, have been proposed to improve the dispersion of CNTs. These techniques are applied in solvent media like water or in polymer media [94]. It has been suggested that CNTs can be hydrophilically modified to improve their dispersion and compatibility with polymer matrices. To counteract van der Waals forces via physical or chemical methods, hydrophilic modification is mostly accomplished by adding hydrophilic specific groups to carbon nanotubes (CNTs) as a spatial site resistance. Physical modification techniques such as adsorption or encapsulation of organic molecules around carbon nanotubes by hydrophobic or van der Waals interactions, such as low molecular weight surfactants, supramolecules, or polymers, can accomplish this [95,96].

**Figure 13:**
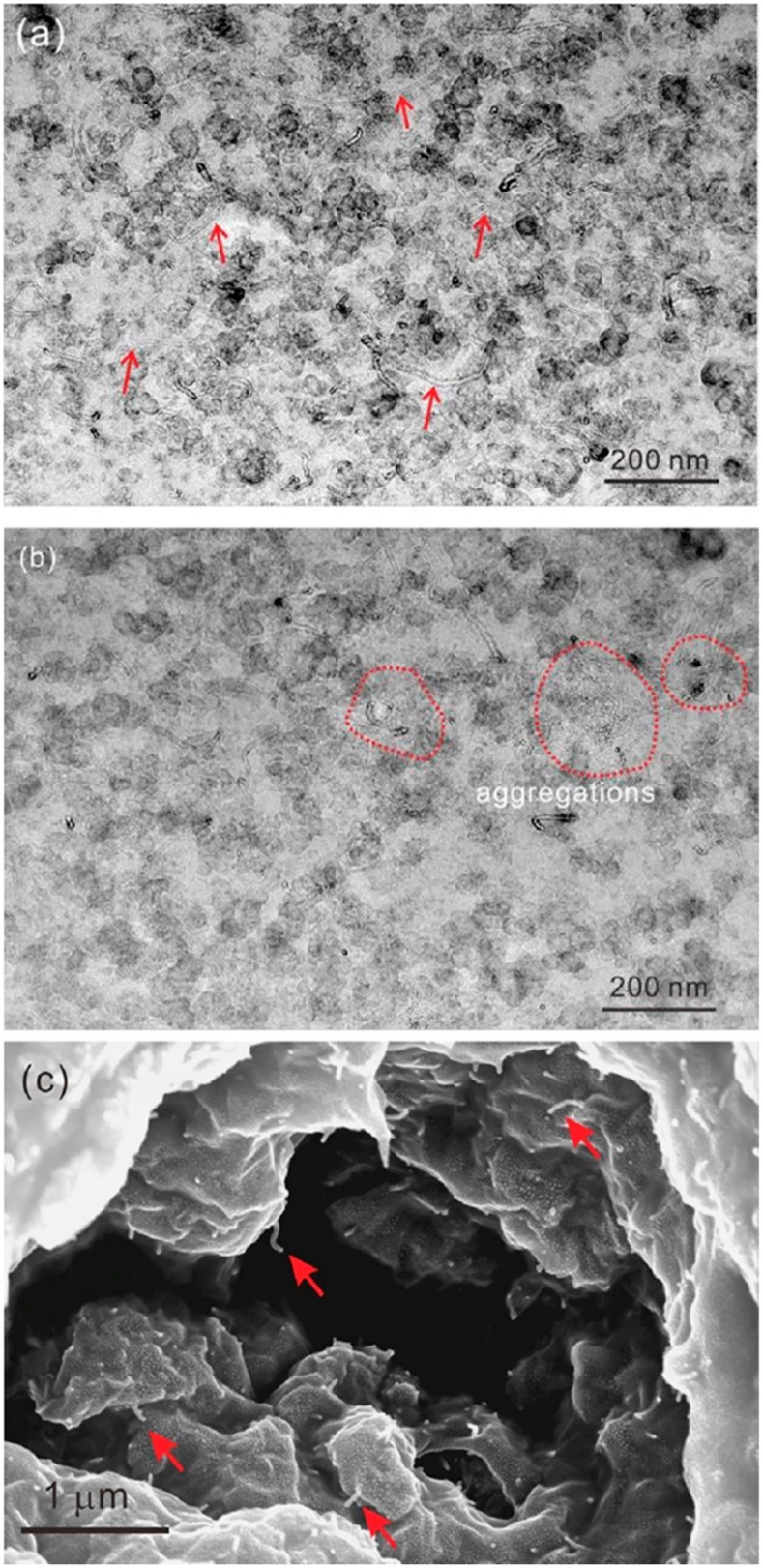
TEM images of NR/CNTs composites prepared by slurry blending method and latex blending method, (**a**) slurry blending method, (**b**) latex blending method. (**c**) The SEM image of NR/CNTs composites prepared by slurry blending method [104].

CNTs/NR composites were prepared by Gao et al. [104] using the slurry mixing method, which decreased carbon nanotube agglomeration and sedimentation when compared to the latex mixing method. This improved carbon nanotube dispersion in NR (Figure 13) led to a 15.2% increase in tensile strength, a more stable thermal conductivity, a weaker Payne effect, and a higher dielectric constant. NR’s interfacial tension was first lowered by SDS, which also enhanced NR’s attraction to CNTs. This enhanced electrostatic driving force between CNTs improved CNT dispersion in the latex. Second, the NR latex became a paste with a decreased interfacial tension as a result of the mixing ingredients increasing its viscosity. The CNTs were evenly distributed throughout the rubber matrix (Figure 13a,c), and the carbon black particles contained a large number of single CNTs (shown by the red arrows in Figure 13). In Figure 13b, some agglomerated CNTs were observed in the red dotted circles. This was most likely caused by adding more filler, which increased the combined latex’s viscosity and decreased its fluidity. Therefore, to prevent subsequent agglomeration, the CNTs were cemented in place after being mixed with NR.

Anand K et al. [106] prepared SWNTs/NR nanocomposites using the latex stage mixing method. After using ultrasonication to disperse the SWNTs in water, surfactants were added to stabilize the mixture. Five distinct surfactants were examined for their dispersing power: sodium benzoate, polyvinyl alcohol (PVA), sodium dodecyl sulfate (SDS), sodium dodecylbenzene sulfonate (NaDDBS), and iso-octylphenoxypolyethoxyethanol (disinfectant) (Creighton X-100). The best dispersing active agent was determined to be NaDDBS, with a mass ratio of 1:5. After being combined with NR latex, the stabilized aqueous dispersion of SWNTs was dried and cured. The outcomes demonstrated that the strength and modulus of natural rubber were greatly enhanced by SWNTs. Electroosmotic flow may be routed to the insulating NR matrix at low-weight fractions.

**Figure 14:**
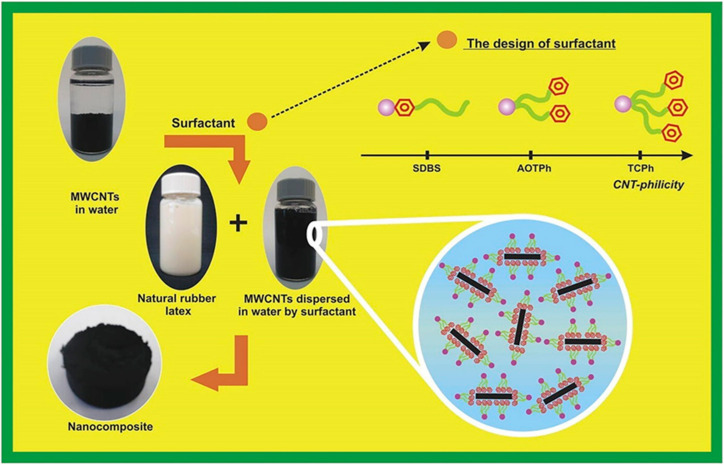
The general route for nanocomposite preparation [102].

Multi-walled carbon nanotube suspension in aqueous solutions of two anionic surfactants (SDS and SDBS) was studied by Bystrzejewski et al. [107]. Both dispersants were shown to be capable of forming stable suspensions of carbon nanotubes below their CMC limits. Compared to SDS, SDBS surfactant had a dispersion capacity that was 26% to 45% higher. While the diameter distribution of CNTs suspended in SDS solution was the same as that of the original CNTs, SDBS demonstrated a larger preference for narrower CNTs. Mohammad et al. [102] used the emulsion mixing approach to treat surfactants and nanocomposites non-covalently, resulting in the successful preparation of homogeneously dispersed MWCNTs in NR latex (Figure 14). First, the surfactant solution was agitated for one hour using five weight percent MWCNTs, followed by three hours of sonication and dispersion. Subsequently, NR latex was introduced to enhance mixing and sonication. To obtain the composites, the materials were finally dried at 80 °C in an oven. The findings demonstrated that, in contrast to the commercially available SDBS surfactant with only one phenyl group, the application of a three-chain surfactant with three phenyl groups significantly stabilized the colloidal mixes of MWCNTs distributed in the NR latex matrix. Furthermore, advantageous π–π interactions between the surfactant tail chain and the MWCNT surface are produced by increasing the number of phenyl rings in the surfactant tail chain from one to three.

The effectiveness of single-, double-, and triple-chain anionic sulfosuccinate surfactants for the dispersion of multi-walled carbon nanotubes (MWCNTs) in natural latex (NR-latex) was examined by Mohamed [97]. Hyperbranched triple-chain sulfosuccinate anionic surfactants, specifically sodium 1,4-bis(neopentyloxy)3-(neopentyloxycarbonyl)-1,4-dioxobutane-2-sulfonate (TC1_4_), were utilized to effectively disperse MWCNTs in NR-latex. This method outperformed sodium dodecyl sulfate (SDS), a commercially available surfactant.

Carbon nanotube-filled natural rubber composites were created by Nakaramontri et al. [98] using melt mixing and latex mixing techniques, respectively. Furthermore, in situ, functionalization of carbon nanotubes using bis(triethoxysilylpropyl) tetrasulphide (TESPT), a silane coupling agent, was conducted to enhance the filler–rubber interactions between rubber molecules and carbon nanotube surfaces. The characteristics of NR–CNT composites are improved, and CNT dispersion is improved with in situ functionalization of CNTs through latex blending and TESPT.

Using Vulcastab VL (poly(ethylene oxide) condensate) as a nonionic surfactant, George et al. [99] were able to produce stable aqueous dispersions of multiwalled carbon nanotubes (MWCNTs). Emulsion mixing assisted by ultrasonography was used to make MWNR/CNTs composites. The best ratio of CNT-to-VL was found to be 1:1, and the lowest amount of ultrasonic energy needed to produce a stable MWCNT aqueous dispersion was 85,000 J.

Multi-walled carbon nanotubes (MWCNTs) were modified, and Ge et al. [100] examined the impact of the hydrophilic ionic liquids 1-ethyl-3-methylimidazolium bromide and 1-hexyl-3-methylimidazolium bromide on this process. After a month of ultrasonic treatment, the carbon nanotubes (CNTs) that were suspended in water remained evenly dispersed. Using a liquid latex mixing technique, high-performance composites comprising natural rubber latex (NRL) and carbon nanotubes modified by IL were created. The NRL/CNT—IL composites’ fatigue resistance and mechanical qualities were enhanced by the homogeneous distribution of CNTs within the matrix.

Multi-walled carbon nanotubes (MWNTs) were oxidized by Xue et al. [101] using potassium persulfate (K_2_FeO_4_) in both neutral and acidic environments. A wet flash evaporation method was used to create the composites of natural rubber, MWNTs, and carbon black. The qualities of rubber products were finally improved by the rise in the oxidation degree of MWNTs, which also raised the number of oxygenated hydroxyl groups on the surface and encouraged their dispersion in the natural rubber matrix. The natural rubber created a suitable route for electron transport, while the effective dispersion of MWNTs decreased the electron transport barrier. According to the experimental findings, K_2_FeO_4_ performed best in an acidic environment and had the maximum degree of oxidation. In comparison to the untreated MWNTs rubber composites, the oxidized MWNTs rubber composites had a 34.9% reduced Payne effect, 15.7% lower wear, 12.7% lower rolling resistance, and 2 orders of magnitude higher electrical conductivity.

**Figure 15:**
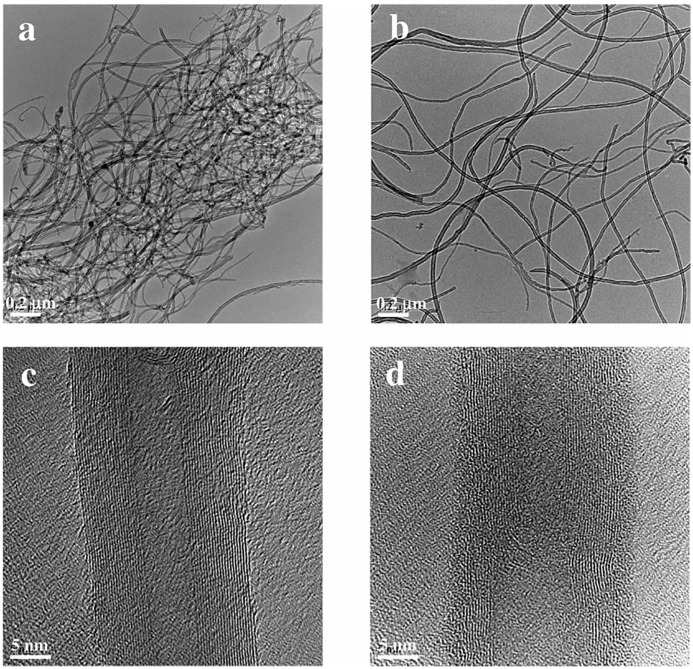
TEM and HRTEM images of aqueous dispersions of (**a**,**c**) pristine MWNTs and (**b**,**d**) SLS-functionalized MWNTs [108].

Liu et al. [108] functionalized multi-walled carbon nanotubes (MWNTs) with sodium lignosulfonate (SLS). A straightforward physical milling method (Figure 15) was able to evenly scatter the SLS-functionalized MWNTs in water, and the MWNTs’ equivalent solubility reached 1.5 mg/mL. Furthermore, the high stability was sustained for over three months. Methylcellulose (MC) was employed by Du et al. [87] to stabilize MWCNT dispersions at a constant mass concentration of 0.08 weight percent. The outcomes demonstrated that MC could considerably raise MWCNT viscosity through the viscosity effect. The findings demonstrated that MC could dramatically improve the stability of MWCNT dispersions in an alkaline environment and significantly increase MWCNT dispersion through the viscosity effect. The addition of 0.18 weight percent MC to MWCNT suspension had the greatest impact on improving MWCNT dispersion.

MWCNT/NR composites were made by Peng et al. [103] utilizing self-assembly and latex mixing methods. After being dissolved in water, the acid-treated MWCNTs and SDS were subsequently added dropwise to the negatively charged latex, dried, and combined. According to the results, MWCNTs were uniformly dispersed in NR latex as single carbon nanotubes, and their surface functionalization significantly inhibited the strong self-aggregation of MWCNTs. Additionally, the uniformly dispersed MWCNTs significantly improved the tensile strength of NR with better endogenous thermal and thermal stability, meaning that only 1 weight percent of the material was used.

To create CB/CNT/NR composites, Zhan et al. [105] also used an ultrasound-assisted latex mixing technique. The outcomes demonstrated that, in comparison to the traditional mixing approach, the ultrasound-assisted latex mixing procedure produced a more uniform dispersion of CNTs in the matrix, and the well-dispersed CNTs and CBs had a synergistic impact. Maximum mechanical characteristics were achieved at a mass ratio of 20:5 (per 100 g of rubber) for CB/CNTs.

To sum up, surfactants can be added or modified to distribute carbon nanotubes evenly throughout the rubber matrix. It is still possible to get a greater reinforcing effect with fewer filler elements. Through functional group modification and ultrasonication, carbon nanotubes’ huge aspect ratio and strong van der Waals force can enhance aggregation.

### 3.4. Graphene (GO)/NR Composites

#### 3.4.1. Mechanism of GO Reinforced Rubber

**Figure 16:**
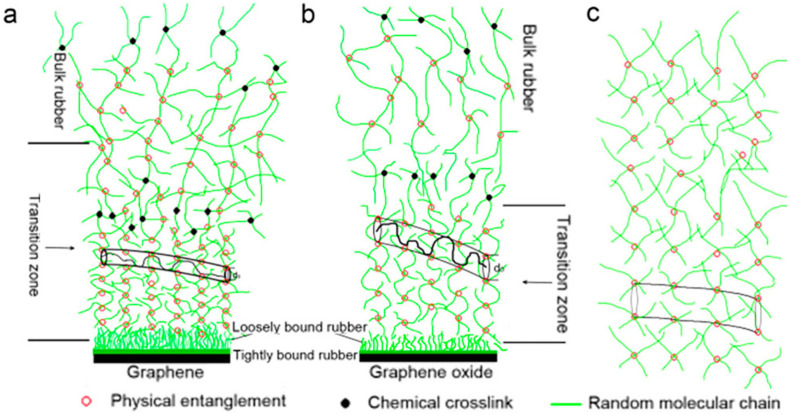
Schematic representation of the EBT model, (**a**) GE/NR (**b**) GO/NR (**c**) NR [109].

Li et al. [109] used the latex mixing method to manufacture GE/NR and GE/NR composites. The in situ chemical reduction method in the latex allowed for the conversion of GO to GE. Additionally, the enhancement mechanism was investigated using the entangled tube model (Figure 16), and the findings indicated that in contrast to the carbon black-reinforced rubber model, GE or GO nanosheets with high specific surface area and nanoscale surface roughness have a greater chance of interacting with the rubber. Due to the high aspect ratio of GE or GO nanosheets, even a tiny quantity of nanolayer dispersion is sufficient to interact with the rubber matrix, allowing only a small number of rubber chains to stay free. Compared to GO, GE is more likely to interact with rubber.

The microstructure and enhancement mechanism of nanocomposites containing graphene nanoparticles (GNPs) in natural rubber (NR) were examined by Li et al [110]. They discovered that the GNPs were folded and annular in the NR matrix and that multiple stacking of the nanoparticles was occasionally visible. Perhaps as a result of heat pressing during processing, the GNPs stayed intact and aligned parallel to the surface of the nanocomposites. The stress-induced Raman Pu band shift was used to quantify the stress transfer from natural rubber to graphene nanosheets.

Although graphene has a stronger reinforcing impact, its potential is still restricted by its aspect ratio, surface roughness, and other parameters, as indicated by the examination of the aforementioned process. Currently, research into the process by which graphene reinforces natural rubber is still needed to fully utilize graphene’s reinforcing properties in rubber.

#### 3.4.2. Graphene/NR Composites Wet Mixing Preparation Process

Graphene has garnered significant interest as a rubbery composite material due to its exceptional electrical, thermal, and mechanical properties [111]. As a result, there has been a gradual increase in wet preparation investigations (Table 5). Nevertheless, it is difficult and expensive to produce clean graphene on a big scale. Modifying graphene can prevent its uniform dispersion in the polymer matrix, which is still a significant issue. Three modes of analysis were used by Hassanzadeh-Aghdam et al. [112] to examine the impact of graphene dispersion on the creep properties of nanocomposites: agglomeration, homogeneous dispersion, and directional alignment. The results showed that the highest creep modulus was achieved by aligning GNPs, while the lowest was achieved by agglomeration. An increased creep resistance is a result of the interfacial phase’s effect when its mechanical properties surpass those of the matrix. Compared to the randomly distributed nanocomposites, the orientated aligned GNPs nanocomposites showed superior creep properties.

Hydrophilic groups in the structure of graphene oxide (GO), an oxidation product of graphene, facilitate its dispersion in aqueous solutions. Graphene oxide may be obtained in vast quantities at a lower cost than pristine graphene. Furthermore, GO has excellent mechanical characteristics and a large concentration of oxygen functional groups. When all these characteristics are combined, GO becomes a more potent reinforcing agent [122]. Reduced graphene oxide (rGO) is a structure that is created when reduced graphene is stripped of its oxygen-containing groups [123]. This is one of the derivatives of graphene, which are also known as reduced graphene, functionalized graphene, chemically modified graphene, and chemically converted graphene [124].

Zhan et al. [113] used in situ reduction techniques and ultrasonic assistance to manufacture NR/graphene (GE) composites. To create the NR/GE masterbatch, graphene oxide was distributed in NRL using ultrasonic, then in situ reduction and latex solidification. In comparison to traditional direct mixing, the technique improved GE’s dispersion and exfoliation from the matrix and enhanced tensile strength, according to the data. With an increase in GE content, the NR/GE composites’ maximum torque, crosslink density, modulus of elasticity, and thermal conductivity all increased.

To create cross-linked rubber composites with electrical conductivity, a water vapor barrier, and high tensile qualities, Zhan [114] et al. used self-assembly and static vulcanization in latex. The composites’ electrical conductivity and electrical impermeability threshold were both significantly increased by the graphene network’s creation. Five orders of magnitude more electrical conductivity was observed in the composites made using the self-assembly procedure in rubber latex with a GE content of 1.78 Vol% as compared to the composites made using conventional methods.

To manufacture innovative graphene/natural latex (NRL) composites, Li et al. [115] developed a stabilizer-free modification technique in which ammonium, the stabilizer, was dispersed during the final heat vulcanization process. The findings demonstrate the viability of the ammonium-assisted approach for the manufacture of graphene/natural latex composites and the superior qualities of the resulting materials. Strong p–p interactions and hydrogen bonding exist between graphene and NRL matrix even at modest loading because of the material’s unique two-dimensional structure and good dispersion.

GO/NR nanocomposites were created by Stanier et al. [116] by combining NR latex with an aqueous dispersion of GO flakes. Rectangular specimens underwent tensile tests at various strain rates up to 600% distortion. Even with modest filler content, the tensile tests demonstrated a considerable rise in the composites’ Young’s modulus, confirming the strong interfacial contact between GO and natural rubber. As the GO content rises, Young’s modulus rises with strain rate, and dissipation rises as a result of higher friction brought on by the high aspect ratio GO. These results indicate that, in comparison to the widely utilized carbon black or carbon nanotubes, considerable increases in mechanical characteristics can be obtained by employing smaller quantities of fillers.

Suriani et al. [117] created GO/NRL nanocomposites in a single step by combining GO and NRL in a 1:1 volume ratio. They also generated surfactant SDS at a concentration of 0.01 M by electrochemical stripping. The GO/NRL nanocomposites prepared in one step (103.7 Fg^−1^) and the nanocomposites prepared in two steps (32.6 Fg^−1^) had a lower specific capacitance. This method involved preparing the GO/NRL nanocomposites concurrently with the GO and NRL mixing product. Consequently, the synthesized GO/NRL nanocomposites may find use in energy storage devices as supercapacitors.

Sodium poly 4-styrene sulfonate was utilized by Li et al. [118] as a stabilizer for the chemical reduction of graphite oxide. The resulting graphene was then combined with NR using a latex process. It was demonstrated that adding surfactants to the graphene surface causes NR molecules to experience both spatial site resistance and electrostatic repulsion. Using the latex composite approach, high-performance NR/graphene nanocomposites were created in conformity with the morphological findings, and the generated nanocomposites’ energy storage modulus was greatly increased.

Following the solidification and drying of rGO/NR latex mixtures made by ultrasound-assisted latex mixing and in situ reduction, Wang et al. [119] developed rGO/CB/NR composites. Both rGO and CB were evenly distributed throughout the rubber composites, as demonstrated by morphological observations. The inclusion of rGO significantly increased the composites’ hardness, heat conductivity, and resistance to aging. When compared to normal latex mixing, the rGO/CB/NR composites made with ultrasonic assistance had superior mechanical characteristics.

Mao et al. [120] created GO/NR composites by using the latex co-coagulation process. By ultrasonically removing graphite oxide from water, combining the aqueous GO dispersion with the latex, and co-coagulating the mixes with the addition of a flocculant, an aqueous GO dispersion stabilized by electrostatic repulsion was produced. The outcomes demonstrated the strong interfacial contacts between GO and NR and the fine dispersion of the highly exfoliated GO flakes in the NR rubber matrix. The mechanical characteristics of the GO/NR composites were further assessed by the researchers. With a GO concentration of less than 2 phr, the results demonstrated a considerable improvement in the tensile strength, rip strength, and modulus. In particular, because of the stress-induced crystallization effect of NR, GO shows a unique strengthening mechanism in NR.

Using the latex co-precipitation approach, Li et al. [121] created CB/GO/NR composites with various crosslinked networks. In the meantime, research was done on how various crosslinking techniques affected fatigue life and crack extension resistance in various vulcanization systems. The findings demonstrated that, in the conventional vulcanization (CV) system, the polysulfone-based CB/GO/NR composites had the lowest crack extension rate (64.1 nm/cycle) and the highest tear strength (71.6 KN/m). The improvement in crack extension resistance was primarily attributed to the CV system’s well-developed crosslinking network and polysulfone-based crosslinking structure.

A wide range of graphene can be modified to improve its dispersion in a rubber matrix or water. With the emergence of several of the aforementioned surfactants and processes, graphene dispersion acts as a potent aid, and when compared to conventional dry mixing, wet mixing can better capture the graphene’s reinforcing properties.

## 4. Expectations and Conclusions

Due to its distinct benefits over dry mixing, wet mixing technology has drawn a lot of attention and has recently been a hot topic for research. The review above indicates that the primary issue with CB/NR composites is the high density and hydrophobicity of carbon black, which settles a lot in the aqueous dispersion and makes it difficult to co-settlement well with the latex. This requires modification with a dispersant or surfactant. The effective interface between particular active sites on the surface of carbon black and the rubber macromolecular chains is always reduced or hindered by the surface’s preferential contact with and adsorption of surfactants.

A significant amount of research has also been conducted on wet mixing technology for SiO_2_/NR composites. However, because of the conflict between silanization and the rubber material’s characteristics and the high viscosity of the prepared masterbatch resulting from the presence of SiO_2_, overloading the mixing equipment can easily occur, impeding appropriate mixing and processing. Wet mixing methods for graphene/NR and NR/CNT composites have been extensively studied, and related research is still ongoing.

In summary, weakening filler–filler interactions and strengthening filler–matrix interactions are necessary to greatly improve the characteristics of NR nanocomposites. However, the broad use of NR composite nanomaterials is restricted by the weaker interactions between nonpolar NR and other nanofillers. As this review points out, better dispersion mixing techniques and filler surface functionalization can help with this. In summary, the wet mixing method makes up for the drawbacks of conventional dry mixing and is anticipated to become a widely used emergent technology.

## Figures and Tables

**Table 1:** Carbon black water dispersion main method.

Form	Methodologies
1	Using water-dispersible amphiphilic polymer structures or traditional surfactants to disperse carbon black in water
2	Carbon black suspension in water, precipitation encapsulation, and emulsion polymerization
3	Methods like using the right oligomers to ball mill carbon black powder in the solid state

**Table 2:** Summary of modified silica/natural rubber composite methods improved in this paper.

NR Composites	Modified Treatment Method/Reagent	Preparation Method	Year/Reference
NR/SiO_2_	Methyltriethoxysilane, vinyltriethoxysilane, γ-aminopropyltrimethoxysilane	Sol-gel method	2014 [51]
NR/SiO_2_/GE	Electron-beam irradiation	Latex mixing	2019 [52]
NR/SiO_2_	Deep eutectic solvents (DES) 3phr	Mechanical mixing	2022 [53]
NR/SiO_2_	Cystamine dihydrochloride (CDHC)	Latex mixing	2020 [54]
NR/SiO_2_	AEO-9, KH-590	Solution compounding	2022 [6]

**Table 3:** Summary of wet preparation methods for silica/rubber composites.

NR Composites	Synthesis	Flocculants/Methods	Year/Reference
NR/SiO_2_	Latex compounding self-assembling techniques	MgSO_4_	2019 [55]
NR/SiO_2_	Latex co-coagulation method	3 wt% acetic acid	2020 [56]
NR/SiO_2_	Latex co-coagulation method	Calcium chloride/acetic acid	2020 [57]
NR/ENR/SiO_2_	Wet masterbatch technique	Ethanol	2016 [58]
NR/SiO_2_	Solution compounding	Solvent removal	2022 [6]
NR/SiO_2_	Latex co-coagulation method	Calcium chloride	2011 [59,60]
NR/SiO_2_	Latex co-coagulation method	Acetic acid	2022 [61]

**Table 4:** Summary of methods mentioned in this paper on wet preparation of NR Carbon Nanotube composites.

NR Composites	Synthesis Method	Surfactants/Modifiers	Year/Reference
NR/MWCNT	Latex co-coagulation method	TCl_4_	2014 [97]
NR/CNT	Latex co-coagulation method	SDS	2016 [98]
NR/MWCNTs	Latex co-coagulation method	Vulcastab VL	2017 [99]
NR/MWCNTs	Latex co-coagulation method	1-ethyl3-methylimidazolium bromide and 1-hexyl-3-methylimidazolium bromide	2018 [100]
NR/MWNTs	Latex co-coagulation method	K_2_FeO_4_	2024 [101]
NR/MWCNTs	Latex co-coagulation method	SDBS/AOTPh/TCPh	2015 [102]
NR/SWNTs	Latex co-coagulation method	NaDDBS	2009 [102]
NR/MWCNTs	Latex co-coagulation method	SDS	2010 [103]
NR/CNTs	Slurry blending method	SDS	2019 [104]
NR/CB/CNTs	Latex co-coagulation method	Emulsifier OP	2011 [105]

**Table 5:** Summary of methods mentioned in this paper on wet preparation of NR/graphene composites.

NR Composites	Dispersal Methods	Improvement of Performance	Year/Reference
NR/GE	Ultrasonic dispersion	Mechanical properties	2011 [113]
NR/GE	Ultrasonic irradiation	Electrical conductivity	2012 [114]
NR/graphene	Ultrasonic irradiation	Mechanical properties	2013 [115]
NR/GO	Vibrate	Mechanical properties	2014 [116]
NR/GO	Mechanical stirring and bath	Electrical conductivity	2015 [117]
NR/rGO	Mechanical stirring	Electrical conductivity Thermal conductivity	2017 [118]
rGO/NR/CB	Ultrasonic dispersion	Mechanical properties	2018 [119]
NR/GO	Ultrasonic dispersion	Mechanical properties	2020 [120]
CB/GO/NR	Ultrasonic dispersion	Mechanical properties	2023 [121]
